# Novel Cascade Alpha Satellite HORs in Orangutan Chromosome 13 Assembly: Discovery of the 59mer HOR—The largest Unit in Primates—And the Missing Triplet 45/27/18 HOR in Human T2T-CHM13v2.0 Assembly

**DOI:** 10.3390/ijms25147596

**Published:** 2024-07-11

**Authors:** Matko Glunčić, Ines Vlahović, Marija Rosandić, Vladimir Paar

**Affiliations:** 1 Faculty of Science, University of Zagreb, 10000 Zagreb, Croatia; vpaar@hazu.hr; 2 Department of Interdisciplinary Sciences, Algebra University College, 10000 Zagreb, Croatia; ines.vlahovic@algebra.hr; 3 University Hospital Centre Zagreb (Ret.), 10000 Zagreb, Croatia; rosandic@hazu.hr; 4 Croatian Academy of Sciences and Arts, 10000 Zagreb, Croatia

**Keywords:** orangutan, complete genomic assembly, alpha satellites, higher-order repeats HORs, human centromere, GRM2023 algorithm

## Abstract

From the recent genome assembly NHGRI_mPonAbe1-v2.0_NCBI (GCF_028885655.2) of orangutan chromosome 13, we computed the precise alpha satellite higher-order repeat (HOR) structure using the novel high-precision GRM2023 algorithm with Global Repeat Map (GRM) and Monomer Distance (MD) diagrams. This study rigorously identified alpha satellite HORs in the centromere of orangutan chromosome 13, discovering a novel 59mer HOR—the longest HOR unit identified in any primate to date. Additionally, it revealed the first intertwined sequence of three HORs, 18mer/27mer/45mer HORs, with a common aligned “backbone” across all HOR copies. The major 7mer HOR exhibits a Willard’s-type canonical copy, although some segments of the array display significant irregularities. In contrast, the 14mer HOR forms a regular Willard’s-type HOR array. Surprisingly, the GRM2023 high-precision analysis of chromosome 13 of human genome assembly T2T-CHM13v2.0 reveals the presence of only a 7mer HOR, despite both the orangutan and human genome assemblies being derived from whole genome shotgun sequences.

## 1. Introduction

Recent dramatic advances in long-read sequencing, coupled with innovations in read length and accuracy, have facilitated the generation of complete human chromosome assemblies, such as T2T-CHM13 [1,2,3,4,5,6,7]. Until recently, the centromeric region of the human genome remained largely uncharted, resembling a “black hole” that restricted the study of centromere organization and function, as well as the complete human genome. This limitation has had implications for both health and disease. Notably, complete sequencing has spurred studies focusing on higher-order repeats [8].

However, recent complete genomic studies of human and selected non-human primate centromeres have revealed their “unimaginable diversity and speed of evolutionary change and the complexity of their genomic organization,” which had been almost impossible to study before [1,9]. In complete genomes of primates, human centromeres are among the most diverse and rapidly evolving regions of the genome [10,11].

Based on the limited sequencing data available since the 1980s, it was discovered that human centromeres contain approximately 171 base-pair alpha satellite repeat monomers. These monomers are organized into sequences of *n* monomers, referred to as *n*mer HORs [12,13,14,15,16,17,18,19,20,21,22,23,24,25]. The divergence between monomers within each HOR copy was found to be significant, ranging from approximately 20% to 40%. HOR copies are further organized in tandem, so that divergence between HOR copies is typically less than 5%. Monomers with a mutual divergence of less than 5% were classified as belonging to the same monomer type. Willard and colleagues discovered that, within each HOR copy, all constituent monomers belong to different monomer types. This pattern, known as Willard’s-type HORs, has been extensively studied using the sequencing data available at the time [26,27,28,29,30,31,32,33,34,35,36,37,38].

In a Willard’s-type *n*mer HOR array, the most common HOR copy, consisting of *n* monomers, is referred to as the canonical copy. Copies within the same HOR array that contain inserts or deletions relative to the canonical HOR copy are called variants. It should be noted that the identification and characterization of HORs within a given genomic sequence present a highly intricate computational challenge, requiring sensitive approximations. This task is further complicated by the significant limitations of earlier sequencing technologies. Various algorithms are available for identifying higher-order periodicities within genomic sequences (e.g., [39,40,41,42,43,44,45,46,47]), highlighting the computational complexity of the problem.

It was found that the orangutan alpha satellite HOR arrays are organized as a “mosaic patchwork” of distinct alpha satellite HOR blocks with a high degree of divergence [9,33,48,49,50].

Here, for the first time, we determine the explicit form of the superHOR structure in the most recent genomic assembly of the orangutan using our novel HOR searching algorithm, which is applicable to HORs with complex monomer repeats.

The Global Repeat Map (GRM) algorithm is a convenient tool for the precise identification of detailed Willard’s-type higher-order repeats (HORs) [21,31,51,52]. It is noteworthy that the GRM algorithm provides a significant advantage by enabling the precise determination of HORs, thereby facilitating the complete identification of both the length and structure of all HOR copies.

This capability was recognized in a study that employed the NTRprism algorithm [4], which is analogous to classical restriction digest experiments but enhanced for the computational analysis of all possible k-mers. NTRprism was able to accurately identify repeat periodicities in alpha canonical HORs across the genome, matching the canonical periodicity in the majority of cases and performing well even on simulated sequences with varying degrees of divergence [4]. By studying the sequence relationships of αSat repeats in detail across each centromere, the study found genome-wide evidence that human centromeres evolve through “layered expansions.” As pointed out by the authors of Ref. [4], NTRprism is similar to the GRM method described in Ref. [53]. However, a limitation of this approach is its design specificity for Willard’s-type HORs, which contain one monomer of each type in canonical HOR copies.

To overcome this limitation, we introduced an updated algorithm named GRM2023 [54], which builds upon and enhances our earlier Global Repeat Map (GRM) algorithm [21,31,53]. GRM2023 extends its scope beyond Willard’s-type HORs, focusing additionally on HORs where monomer types are not all different but rather some are repeated. We term these extended HORs as cascading HORs. This advancement allows for the precise identification of such structures.

A rigorous description of the structural organization of alpha satellite higher-order repeat sequences (HORs) poses a complex challenge, often leading to discrepancies in results obtained using different computational methods. The GRM and GRM2023 tools offer a notable advantage over alternative algorithms by providing high precision in identifying HOR copies and elucidating their structure. The GRM2023 algorithm detects peaks corresponding to alpha satellite HORs, as well as additional peaks representing subfragments and non-tandem repeats.

Our recent studies [54] have applied similar approaches to human chromosome 15, unveiling intricate centromere structures with profound implications. This study revealed novel cascading HORs and highlighted the complexity within the chromosome 15 centromeric region, emphasizing deviations from anticipated highly regular patterns and suggesting significant information-encoding and functional potential within the human centromere.

We also note that the method of mass characterization based on the hydrogen atom content was recently employed in a study examining the symmetries of genetic code classification [55], suggesting a potential extension to HOR symmetries.

## 2. Results and Discussion

### 2.1. GRM (Global Repeat Map) Diagram and MD (Monomer Distance) Diagram for Orangutan Chromosome 13

**Figure 1:**
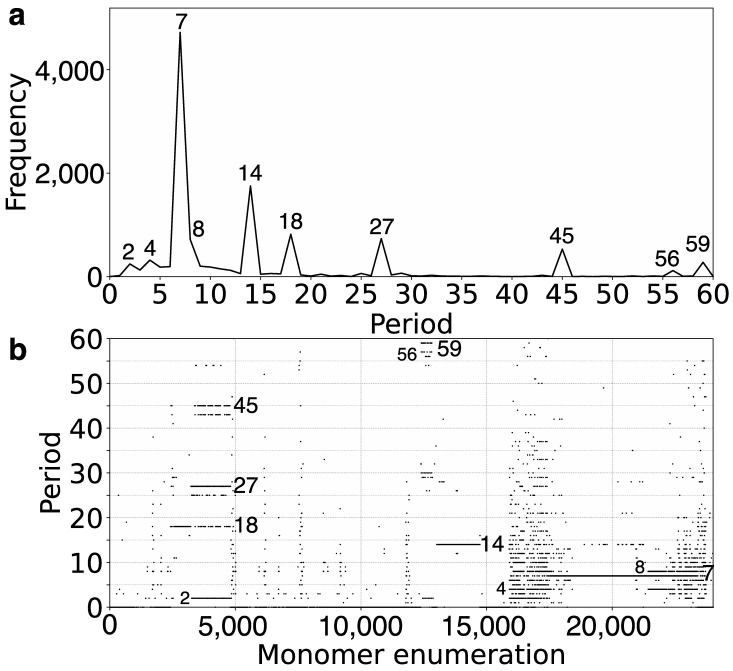
GRM (Global Repeat Map) diagram and Monomer Distance (MD) diagram for tandemly arranged alpha satellite monomers in complete GCF_028885655.2 assembly of orangutan chromosome 13. (**a**) GRM diagram. Horizontal axis: GRM periods. Vertical axis: frequency of monomer repeats period. Identified GRM peaks corresponding to HORs have periods 7, 14, 18, 27, 45, and 59, whereas minor peaks at 2, 4, 8, and 56 correspond to subfragments. (**b**) MD diagram. Horizontal axis: enumeration of tandemly organized alpha satellite monomers. Vertical axis: period (distance between the start of one monomer and the start of the next monomer of the same type). Distinct regions with MD line segments are evident for periods 7, 14, 18, 27, 45, and 59, corresponding to 7mer, 14mer, 18mer, 27mer, 45mer, and 59mer HORs, respectively. Some additional line segments correspond to subfragments of HORs or less pronounced repeats, which will be discussed below. Additionally, there are some randomly scattered MD points.

Here, we utilized the GRM2023 algorithm to investigate the recent genome assembly of orangutan chromosome 13, based on the NHGRI_mPonAbe1-v2.0_pri assembly (NCBI RefSeq assembly GCF_028885655.2), which was generated using PacBio Sequel and Oxford Nanopore PromethION sequencing technologies. Initially, tandemly organized alpha satellite monomers were identified within the genomic assembly of orangutan chromosome 13, enumerated in the order of their appearance in assembly. Using the precise GRM2023 algorithm, we computed the corresponding GRM diagram for this array of tandemly organized monomers. HORs were identified as prominent peaks in the GRM diagram: a peak of period *n* corresponds to *n ×* 171 bp, representing the *n*mer HOR [54]. The GRM diagram for the complete assembly of orangutan chromosome 13 is depicted in Figure 1a. As demonstrated here, the GRM peaks at periods 7, 14, 18, 27, 45, and 59 correspond to 7mer, 14mer, 18mer, 27mer, 45mer, and 59mer HORs, respectively.

The MD (Monomer Distance) diagram depicts periods (vertical axis) as a function of monomer enumeration (horizontal axis) (Figure 1b). Each point on the MD diagram represents a monomer enumeration on the horizontal axis and its corresponding distance to the next monomer of the same type within the sequentially organized monomer sequence, determining its horizontal and vertical coordinates [54]. These points, referred to as MD points, form densely distributed horizontal MD line segments that correspond to an HOR, where the vertical coordinate represents the period of the HOR. For each HOR, these MD points form closely clustered horizontal line segments visually resembling continuous lines in the interval corresponding to monomers constituting HOR. The uppermost MD line segment within a monomer enumeration interval corresponds to the *n*mer HOR array. Observing the MD diagram (Figure 1b and Table 1), the predominant MD line segment for orangutan chromosome 13 corresponds to the 7mer HOR. However, alongside the dominant MD line segment at period 7, other scattered points appear within the same monomer enumeration interval, indicating significant deviations from the regular 7mer HOR pattern. This includes the presence of equidistant or dispersed subfragments, analogous to those observed in the human chromosome as described in Ref. [54].

**Figure 2:**
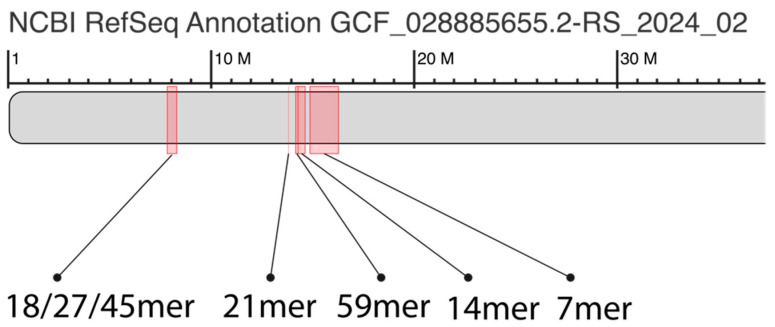
Ideogram of major alpha satellite HOR arrays in the centromeric region of assembly of orangutan chromosome 13. Cascading 18/27/45 HOR array, riddled divergent 21mer HOR array, cascading 59mer HOR array, Willard’s-type 14mer HOR array, 7mer cascading HOR array.

The ideogram (Figure 2) illustrates the spatial arrangement of major alpha satellite HOR arrays identified from the GRM and MD diagrams for orangutan chromosome 13 (Figure 1a,b), highlighting their distribution and relative positions within the centromeric region.

### 2.2. Novel Orangutan Cascading 59mer HOR—The Largest Alpha Satellite HOR Copy Discovered in Primate Genomes

**Figure 3:**
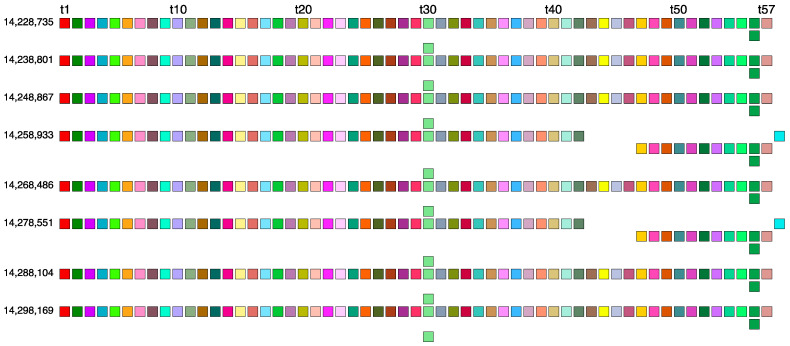
Aligned scheme of a cascading 59mer HOR array consisting of six canonical 59mer HOR copies and two 56mer variants. Different monomer types are indicated by unique colors within their respective boxes. The constituent monomers, designated as t1, t2, t3, … t58, are each distinct from one another. In the MD diagram shown in Figure 1b, the MD line segment corresponds to the canonical 59mer HOR copies (period 59) and a shorter MD line segment corresponds to the 56mer variant (period 56). The two HORs at positions 14,258,933 and 14,278,551 are 56mer variant HORs that differ from the canonical due to a mutation in monomer t43 and a deletion of the triplet of monomers t44-t45-t46. The green monomer types singled out in the second and third row of each HOR copy represent duplications of monomers t56 and t30, respectively, which contribute to the cascading nature of the 59mer HOR array.

In the GRM diagram for orangutan chromosome 13, the GRM peak of the highest period emerges at period 59 (Figure 1a). Correspondingly, in the MD diagram (Figure 1b), a notable MD line segment comprising approximately 500 tandemly organized monomers appears at a monomer enumeration of around 12,400, aligning with the aforementioned GRM peak. Furthermore, analysis using the GRM2023 algorithm reveals the presence of eight cascading 59mer HOR copies, with six canonical 59mer HOR copies, as shown in the aligned graphical presentation in Figure 3.

As seen from the HOR structure diagram, the canonical cascading 59mer HOR copy contains two duplicated monomer types: monomers of the same type 30 in the first and third rows of the HOR copy, and of the same type 56 in the first and second rows. The computed average divergence among monomers in each canonical HOR copy is 22.4%, while the divergence between the two HOR copies is much smaller, only 0.1%, consistent with the characteristic 59mer HOR pattern. The computed MD point frequency 277 (Table 1) corroborates the existence of 59mer HOR copies. This 59mer HOR copy represents the largest canonical HOR unit identified to date in all primate genomes. The consensus monomers in the canonical 59mer HOR copy are presented in Supplementary Table S1.

The canonical 59mer HOR copy consists of 59 monomers of 57 different types. Consequently, we have a cascading 59mer HOR array composed of six canonical 59mer HOR copies and two of its 56mer variants. In comparison to the canonical HOR copy, the variants have four deleted monomers and one additional monomer of a different type, resulting in a 56mer variant. Thus, the cascading 59mer HOR array pattern is CCCVCVCC, where ‘C’ denotes canonical, and ‘V’ denotes variant copies of the same type of HOR. The consensus sequence of the 57mer canonical HOR copy is provided in Supplementary Table S1.

### 2.3. Aligned Scheme for 7mer HOR Array with 2mer, 4mer, 8mer Subfragments

Based on the graphical representation provided by the GRM and MD diagrams (Figure 1a,b), the largest HOR array within orangutan chromosome 13 is identified as a 7mer HOR. This corresponds to the longest MD line segment with a period of 7 (vertical axis in Figure 1b), extending from monomer enumeration ~17,000 to ~23,000 on the horizontal axis.

**Figure 4:**
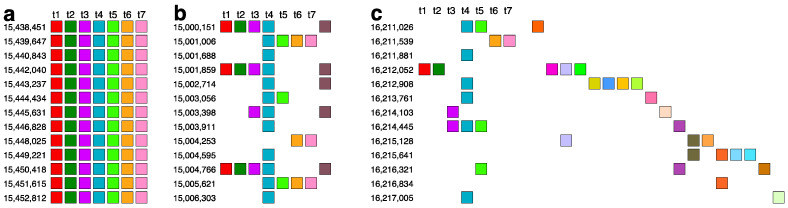
Illustrative segments from 7mer HOR array. (**a**) A segment containing canonical 7mer HOR copies (position from 15,438,451 to 15,452,812) in the central part of 7mer HOR array. Monomers in HOR copies are aligned according to their type. In the top row above each box, the corresponding type is labeled as t1, t2, and so forth. Different monomer types are distinguished by varying box colors, while monomers of the same type share identical coloring. This presentation of the HOR scheme is described in Ref. [54]. (**b**,**c**) Segments (positions from 15,000,151 to 15,006,303 and 16,211,026 to 16,217,005) with increased deletions within the alignment and increased insertions outside the alignment band. In the GRM diagram, the main peak denoted 7 corresponds to the 7mer HOR, and the peaks denoted 8, 4, and 2 correspond to subfragments with periods of 8, 4, and 2, respectively, associated with the 7mer HOR (Figure 5).

The comprehensive alignment pattern of the 7mer HOR array, computed using the GRM2023 algorithm, is depicted in Supplementary Figure S2. Its canonical 7mer HOR copy is of Willard’s type, meaning all seven constituting monomers are of different types. The consensus sequence of the 7mer canonical HOR copy is provided in Supplementary Table S2. As an illustration, a segment of 7mer HOR copies in the central part, consisting largely of canonical 7mer HOR copies, is shown in Figure 4a. The corresponding signature of this canonical HOR pattern is the most pronounced horizontal MD line segment in Figure 1b at period 7 (period on vertical axis).

**Figure 5:**
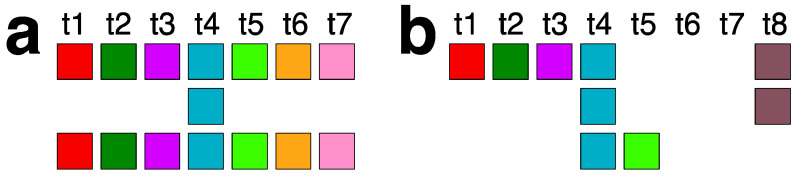
Origin of 8mer, 4mer, and 2mer subfragments in the 7mer HOR array. (**a**) A periodic substructure triplet of three aligned 7mer HOR copies: (t1, t2, t3, t4, t5, t6, t7)—(t4)—(t1, t2, t3, t4, t5, t6, t7). In each substructure, an eight-step periodicity (t1→t2→t3→t4→t5→t6→t7→t4→t1) generates a pattern of period 8. This periodicity is consistent even if initiated from t2, t3, t5, t6, or t7, thereby synchronizing to produce the MD frequency of period 8. Starting from t4, the periodicity (t4→t5→t6→t7→t4) results in a 4mer subfragment structure. (**b**) A 2mer subfragment pattern (t4→t8→t4 and t8→t4→t8) emerges within a periodic substructure of four aligned 7mer HOR copies: (t1, t2, t3, t4, t8)—(t4, t8)—(t4, t8)—(t4, t5).

On both sides of the region dominated by canonical 7mer HOR array copies, deletions within canonical HOR copies increase, insertions become more numerous, and traces of monomer-type alignment gradually disappear, as seen in Supplementary Figure S2. This situation is illustrated by segments of the 7mer HOR copies in Figure 4b,c. In such cases, equidistant repeats appear within the 7mer HOR array corresponding to subfragments. The most pronounced subfragments in the 7mer HOR array are the 8mer, 4mer, and 2mer. In the GRM diagram, the main peak denoted 7 corresponds to the 7mer HOR, and the peaks denoted 8, 4, and 2 correspond to subfragments with periods of 8, 4, and 2, respectively, associated with the 7mer HOR (Figure 5). In addition to canonical 7mer copies, there exists a periodic substructure triplet consisting of three aligned 7mer HOR copies, represented as (t1, t2, t3, t4, t5, t6, t7)—(t4)—(t1, t2, t3, t4, t5, t6, t7) (Figure 5a). These substructures repeatedly appear at the beginning and end of the 7mer HOR array, for example, starting at positions 16,013,745, 16,015,309, and 16,017,871 (Supplementary Figure S2). Within each substructure, an eight-step periodicity (t1→t2→t3→t4→t5→t6→t7→t4→t1) generates a pattern of period 8, leading to an associated equidistant 8mer subfragment pattern. This periodicity is consistent even if initiated from t2, t3, t5, t6, or t7, thereby synchronizing to produce the MD frequency of period 8, as documented in Table 1. Conversely, initiating from t4 in the same periodic substructure triplet (t4→t5→t6→t7→t4) results in a 4mer subfragment structure. Similarly, a 2mer subfragment pattern (t4→t8→t4 and t8→t4→t8) (Figure 5b) emerges within a periodic substructure of four aligned 7mer HOR copies, structured as (t1, t2, t3, t4, t8)—(t4, t8)—(t4, t8)—(t4, t5) at the beginning of the 7mer HOR array. There are also some other less pronounced periods partially present in the monomer enumeration region of the 7mer HOR array, as shown in Figure 1b; these are mostly constituted of distinguishable MD points at different periods.

### 2.4. Aligned Scheme of Cascading Interspersed 18/27/45mer HOR Array

**Figure 6:**
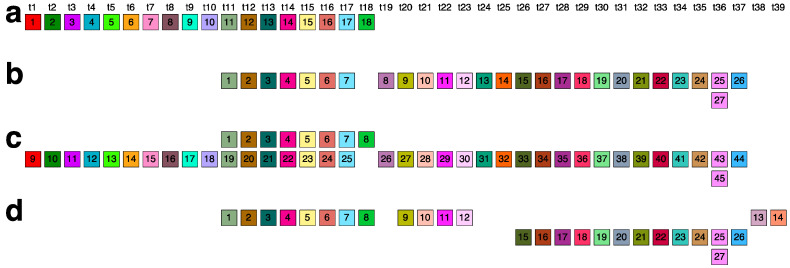
Aligned canonical HOR copies of (**a**) Willard’s-type 18mer HOR, (**b**) cascading 27mer HOR, and (**c**) cascading 45mer HOR. (**d**) Variant of 27mer HOR. Monomers are represented by colored boxes, with each color indicating a specific type. All monomers within the same column are of the same type.

**Figure 7:**
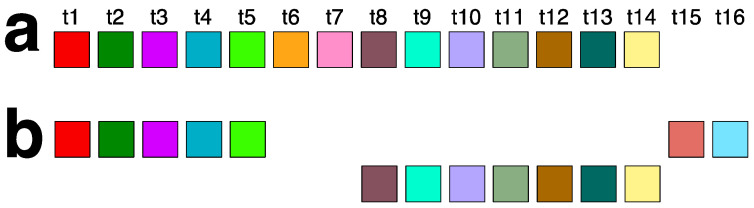
Schemes of canonical and variant 14mer HOR copies. (**a**) Canonical 14mer HOR copy. (**b**) Variant 14mer HOR copies arising from canonical HOR copy by substituting two monomers of types t6 and t7 with monomers of types t15 and t16. This substitution occurs four times in the 14mer HOR array, resulting in four identical variant HOR copies (Supplementary Figure S4).

Using our GRM2023 algorithm, we identified three interspersed HOR patterns: 18mer, 27mer, and 45mer, fully presented in Supplementary Figure S3. Their aligned canonical HOR copy schemes are shown in Figure 6a–c, respectively. The consensus sequence of the 18/27/45mer canonical HOR copy is provided in Supplementary Table S3. The intermixing of the three HOR patterns is summarized in Figure 7. As illustrated, the 18mer HOR copies are almost compact. Additionally, segments of the 18mer are incorporated as structural parts into every 27mer and 45mer copy (Supplementary Figure S3), as illustrated in Table 2.

### 2.5. Aligned Scheme for Willard’s-Type 14mer HOR Array

The 14mer HOR in orangutan chromosome 13 is a highly regular Willard’s type (Supplementary Figure S4). It comprises 131 HOR copies, with 90% of these being canonical 14mer (Figure 7a). Variant HOR copies arise from the canonical HOR by replacing two monomers of types t6 and t7 with monomer types t15 and t16, which differ from monomer types t1 through t14 (Figure 7b). This substitution occurs four times in the 14mer HOR array, resulting in four identical variant HOR copies (Supplementary Figure S4). The consensus sequence of the 14mer canonical HOR copy is provided in Supplementary Table S4.

### 2.6. Aligned Scheme for Highly Riddled Willard’s-Type 21mer HOR Array

**Figure 8:**
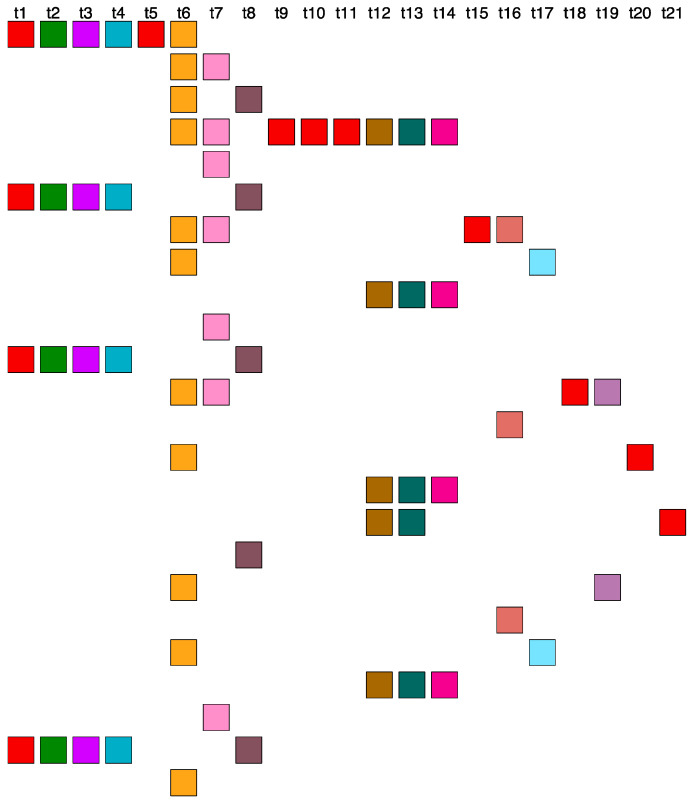
Highly riddled 21mer monomer-aligned HOR-like pattern in the orangutan chromosome 13 assembly.

A highly distorted pattern is observed, comprising monomers aligned in a scheme of 21 different types. The monomers are scattered yet aligned, preventing the identification of a canonical HOR copy (Figure 8). This intricate scheme is characterized by the alignment of monomers according to their types. We emphasize that this is not a true 21mer; rather, it consists of 21 different types of monomers combined into various variant HOR copies, none of which are 21mers, but are instead 19mers, 15mers, and 28mers (Figure 8).

Conclusion: The paradox of exclusively pronounced alpha satellite HORs in the orangutan genome assembly NHGRI_mPonAbe1-v2.0_NCBI (5 January 2024) for chromosome 13, without a counterpart in the human T2T CHM13v2.0 assembly.

**Figure 9:**
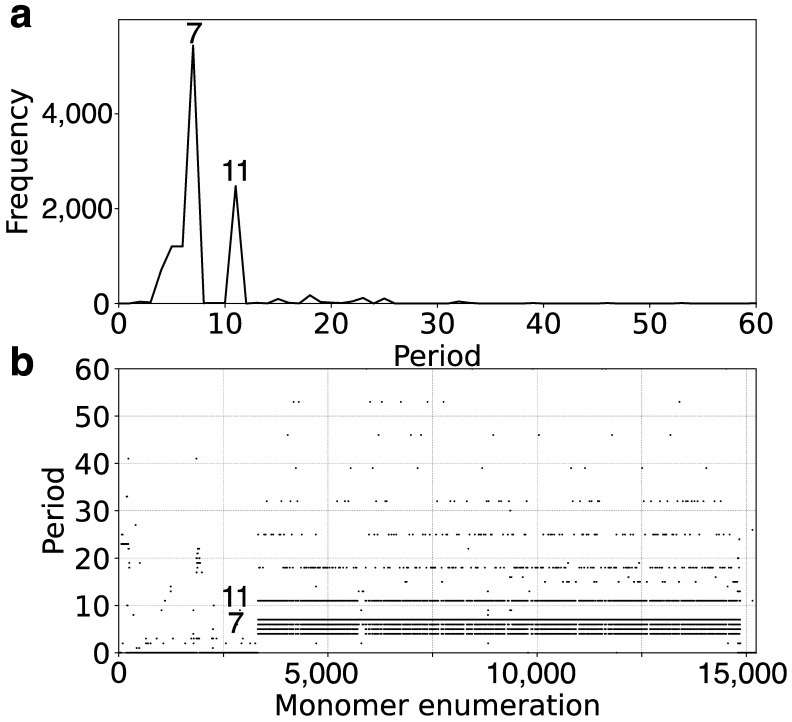
GRM (Global Repeat Map) diagram and Monomer Distance (MD) diagram for tandemly arranged alpha satellite monomers in complete assembly of T2T-CHM13v2.0 human chromosome 13. (**a**) GRM diagram. (**b**) MD diagram.

To compare the recently available orangutan and human alpha satellite HOR arrays, we computed the GRM and MD diagrams for the T2T-CHM13v2.0 assembly of human chromosome 13 (Figure 9a,b). The only pronounced HOR array in the MD diagram of human chromosome 13 is a 7mer HOR within the broad interval of monomer enumeration from approximately 3000 to 15,000. Additionally, subfragments of 4mer, 5mer, 6mer, and 11mer are observed on parallel line segments. The MD diagram reveals that the 7mer HOR array extends over roughly 80% of the monomer enumeration. However, the other pronounced HORs identified in the orangutan genome, such as the 14mer HOR, 18/27/45mer array, and 59mer HOR array, are absent in the genome of human chromosome 13, particularly in the remaining smaller segment of tandem monomer enumeration where no significant HOR array is observed.

On the other hand, in the MD diagram of orangutan chromosome 13, the 7mer HOR array extends over only approximately 30% of the tandem monomer enumeration. In contrast, the remaining nearly 70% of the tandem monomer enumeration consists of significant HOR arrays of the 14mer HOR, 18/27/45mer array, and 59mer HOR array.

Comparing the MD diagrams for human T2T-CHM13v2.0 (Figure 1b) and orangutan (Figure 9b) assemblies reveals that the human tandem monomer range of ~2500–15,000 is similar to the orangutan’s ~15,000–25,000 range. Consequently, the orangutan monomer range of 0–15,000, which includes the 18/27/45mer, 59mer, and 14mer HORs, has no equivalent in the human T2T-CHM13v2.0 chromosome 13 assembly. This is further confirmed by the absence of GRM peaks at periods 14, 18/27/45, and 59 in the human GRM diagram for T2T-CHM13v2.0 (Figure 9a).

This paradox requires further investigation: (a) Are the recently available complete human and chimpanzee assemblies truly complete? (b) Are the chromosome 13 sequences in the human and chimpanzee genomes really so different?

## 3. Methods

Reference genome sequences for orangutan chromosome 13 GCF_028885655.2 are freely available at the National Center for Biotechnology Information official website URL https://www.ncbi.nlm.nih.gov/datasets/genome/GCF_028885655.2/ (accessed on 10 July 2024).

The core tools integrated into the GRM2023 algorithm are our applications MonFinder and GRMhor. Our application, MonFinder (available at github.com/gluncic/GRM2023, accessed on 10 July 2024), was employed to identify alpha satellite monomers within the entire orangutan chromosome 13 GCF_028885655.2 assembly. MonFinder takes genomic sequences (subject) and a consensus sequence (query) as inputs, providing a list of detected monomers. This algorithm utilizes the Edlib open-source C/C++ library for precise pairwise sequence alignment [56]. Within the MonFinder algorithm, the subject sequence is searched in both the direct and reverse complement directions to identify all monomers. In this study, a unique consensus sequence of 171 base pairs (bps) in length (located within the MonFinder code on GitHub San Francisco, CA, USA, github.com/gluncic/GRM2023, accessed on 10 July 2024), derived from over 1,000,000 different alpha satellites across all higher primates, including humans, was utilized as a query for detecting all alpha satellites in the genomic sequence under investigation.

In the subsequent phase, the Python program GRMhor (available at github.com/gluncic/GRM2023, accessed on 10 July 2024) is invoked, taking as its input parameter a file containing a sequence of monomers from the preceding step. Upon loading the monomer array, the application autonomously generates a GRM diagram, MD diagram, and an aligned schematic representation of the monomer organization within the array (Supplementary Figures S1–S4 and Figure 3, Figure 4, Figure 5, Figure 6, Figure 7 and Figure 8 consolidated into a single file).

## Figures and Tables

**Table 1:** Frequency of MD points of different periods ordered by decreasing number of MD points. The major alpha satellite *n*mer HOR array in orangutan is the 7mer HOR, which exhibits a total of 4720 MD points. The second-largest HOR array is the 14mer HOR array with 1753 MD points, followed by the 18mer HOR with 819 MD points, and the 27mer HOR with 734 MD points etc. The cascading 7mer HOR gives rise to a series of subfragments of the 7mer HOR, with the 8mer fragment being the most pronounced.

No. of MD Points	Period	Repeat Pattern
4720	7	7mer HOR
1753	14	14mer HOR
819	18	interspersed 18/27/45mer HOR
734	27	interspersed 18/27/45mer HOR
718	8	8mer subfragment in 7mer HOR
530	45	interspersed 14/27/45mer HOR
319	4	8mer subfragment in 7mer HOR
277	59	59mer HOR
244	2	2mer subfragment in 7mer HOR

**Table 2:** Distribution of HOR copies in the interspersed 18/27/45mer HOR array, starting at position 7,917,393. Marks 18, 27, and 45 denote the canonical 18mer, canonical cascading 27mer, and canonical cascading 45mer HOR copy, respectively. 18v and 45v denote variants of the 18mer and 45mer HORs, respectively. The mark 18’ denotes a variant of 27mer HOR.

1 × 18, 1 × 45v, 1 × 45, 1 × 18v, 33 × 18, 5 × 18v, 1 × 18v, 1 × 27, 5 × 27, 1 × 45, 2 × 27, 1 × 18′, 1 × 45, 1 × 18′,
2 × 45, 1 × 18′, 3 × 45, 2 × 27, 1 × 18′, 1 × 45, 1 × 18′, 3 × 45, 2 × 27, 1 × 18′, 5 × 45, 5 × 27, 1 × 45, 1 × 27, 4 × 45

## Data Availability

The GRM2023 tools MonFinder and GRMhor (python applications) are freely available at github.com/gluncic/GRM2023. Reference genome sequences chromosome 3 GCF_028885655.2 are freely available at the National Center for Biotechnology Information official website https://www.ncbi.nlm.nih.gov/datasets/genome/GCF_028885655.2/ (accessed on 10 July 2024).

## Supplementary Materials

The following supporting information can be downloaded at: https://www.mdpi.com/article/10.3390/ijms25147596/s1.
