# Supplementary material for: Novel Cascade Alpha Satellite HORs in Orangutan Chromosome 13 Assembly: Discovery of the 59mer HOR—The largest Unit in Primates—And the Missing Triplet 45/27/18 HOR in Human T2T-CHM13v2.0 Assembly
Source: Int J Mol Sci. 2024 Jul 11;25(14):7596. doi: 10.3390/ijms25147596 (PMC11276891; doi:10.3390/ijms25147596)
Supplement: Supplementary file 1 [file ijms-25-07596-s001.zip › ijms-3091306-supplementary-1.pdf]

Supplementary Materials for

High precision novel Cascade alpha satellite HORs of orangutan chromosome 13 assembly: discovery of the 59mer HOR - the largest HOR unit in primates, and of intertwined triplet 45/27/18 HOR which are missing in analysis of human T2T-CHM13 assembly

Matko Glunčić, Ines Vlahović, Marija Rosandić, Vladimir Paar

Corresponding author: matko@phy.hr

The Supplementary file includes:

Figures S1 to S4
Tables S1 to S4

Figure S1. Cascading 59mer alpha satellite HOR alignment. Start position 14,228,735 bp and end position 14,308,232 bp in GCF_028885655.2. The numbers on the left side indicate the initial position of the first monomer in each HOR copy.

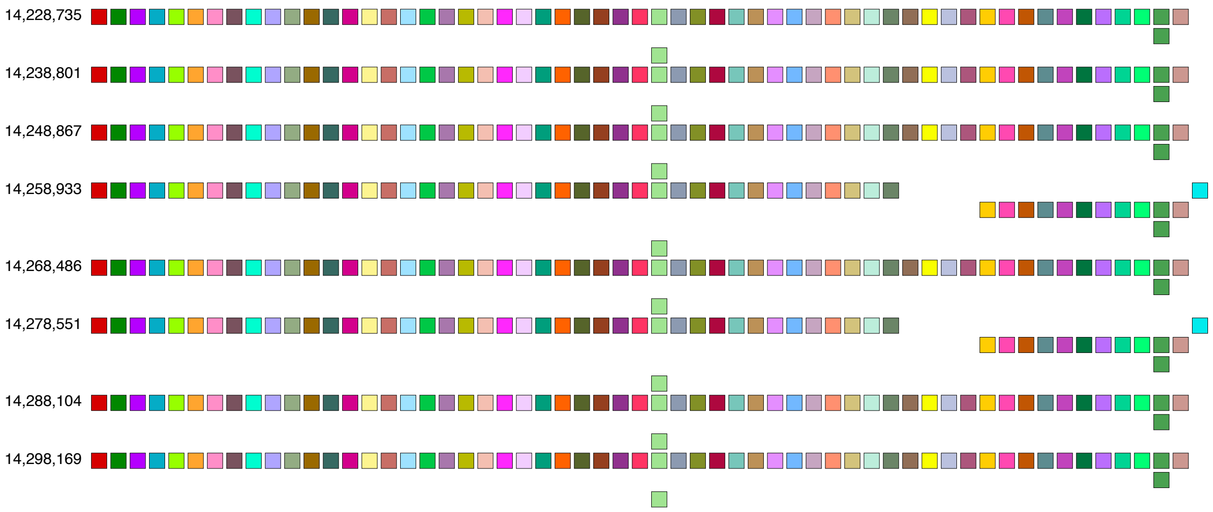

Figure S2. (separate file) 7mer alpha satellite HOR alignment. Start position 14,941,727 bp and end position 16,266,825 bp in GCF_028885655.2. The numbers on the left side indicate the initial position of the first monomer in each HOR copy.

Figure S3. (separate file) Cascading interspersed 18/27/45mer alpha satellite HOR alignment. Start position 7,917,393 bp and end position 8,334,639 bp in GCF_028885655.2. The numbers on the left side indicate the initial position of the first monomer in each HOR copy.

Figure S4. (separate file) Willard's type 14mer alpha satellite HOR alignment. Start position 14,333,149 bp and end position 14,628,690 bp in GCF_028885655.2. The numbers on the left side indicate the initial position of the first monomer in each HOR copy.

Table S1. Canonical 59mer consensus sequence.

Monomer type t1
TCTGTAAATGCTTTGTGTGGTATACATTCAACTCACTGAGTTGAACATTTCTTTTGATAGAT
CAGTTTTGAAACACTGTTTTGTAGAAACTGCAAGTGTTCGTTAGGTGCGCTTTGAGGCCTAT
GGTGGAAAAGGAAATATCTTCACATAAAAACTAGACAGAAACATTC
Monomer type t2
TAAGAAACACCTTTATGATGTGTGCATTCAAGTAACAGAGTTGAACCTTCCTTTTCTTAGAG
CATTTTTGAAACAGTGTTTTTGCAGAATCTGCAAGAGGAAATTTGGAGCTCTGTGAAGCGTT
CGTTGGAAACCTGACTATCTTCACGTAAAACCTAGACAGAAGTGTTC
Monomer type t3
TCAGAAACGGCTTTATGTTGTGGGCACTCGACACACTGAGTTGAACCTTTCTTTTGATAGAG
CACTTTTGAAACATTCTTTTCGTAGAATTTGCAAGTGTTCATTTGGAGTGCTTTGAAGCCTA
CGGTGGAAAAGGAAACAACTTCACATAAAAACTAGACAGAAGCATTC
Monomer type t4
TCTGAAAGTTCTTTATGATGTGTGGTTTCAACTCACAGAGTTCAAACTTCCTTTTGATGGAG
CAATTTTGAAAGGGTCTTTTTGCAGGATCTACAGGTGGATATTTGGAGCTCTTAGAGGCCTC
CGTTGGAAACGTGAATATCTTCACATAAAAACTAGACAGAAGGATTC
Monomer type t5
TAAAACTTCTTTGTGTAGTGTGAATCCAACTCACACACATTTGAACACTTCTTTTAAAAGAG
CAGTTTTGAAACACTCTTTCTGTAGAATCTGCAATTGTTCTTTAGGAGCACTTTGATTCCTA
TTGTGGAAAAGGAAATATCTTCACATAAAATCCAGACAGAAGCATTC
Monomer type t6
TCAGAAACATCTTTATGATGTGTGCATTGCACTCACCGAGTTGAACCTTTCTTTTGATAGAG
CAGTTTTGAAAGAGTGTTTTTGCAGGATCTGCAAGTGGATATTTGGAGCTCTTTGAGGCCTT
CGTTAGAAACGGGAATATCTTGACATAAATCCAGAAAGAAGCATTCTG
Monomer type t7
TCTGAAACTGCTTTGTATTCTATGAACTCAACTCACAGAGTTGAATCTTTCTTTTGATAGAG
CAGTTTTGAAACACTTTTTTGTAGAATTTGCATGCTTTCATTTGGTTCGCTTTGAGGCCTAT
GGTGGAAAAGGAAATATCTTCACATAAAAACTAGAGAGAAGCATTC
Monomer type t8
TCAGAAACAACTTTATGATGTGCGCATTCAACTCAGACAGATAAGCCTTCCTTTTGATAGAA
CAGTTCTGAAACAGAGTTTTTACAGAATCTGCAAGTGGATATTTGGAGCTCTTGAGGTCTTC
GTTGGAAATGGGAATATTTTCACAAAAAAACTAGACAGAGGTGTTC
Monomer type t9
TCTGAAACTGCTTTGTGTTGTATGCATTCAACTCACAGAGTTGAACCTTCCTTTGGATAGAG
CAGTTTTGAAACACCCTTTTTGTAGAATCTACAAGTGTTCATTTGGAGGGCTTTGAGGTCTA
TGGTGGAAAAGGAAATATCTTCACATAACAAATAGACAGAAGCATTC
Monomer type t10
TCTGAAACATCCTTTTGATGTTTGCATTCAACTTACAGACTTGAACCTTCCTTTTCATGCAG
CAGTTTTGAAAGAGTCTTTTTCCAGAATCTGCAAGTGGATATTTGGAGTTCTTTGAGTCCTT
CGCTGGAAACGGGAATATCTTCACAAAAAAACTAGACAGAAGCATTC
Monomer type t11
TGAGGAACAGCTTTGCAATGTGTGGGTTAAACTCACAGAGTTGAATCTTTCTTTAGTAGAGC
AGTTTTGAAAAACTCTTTTTGTAGAACCTGCAAGTGTTCATTTGGTGCGCTTTAAGGCCTAT
GGTGGAAAAGAAAATATCTTCACATAAAATCTAGACAGAAGCATTC
Monomer type t12
TAAGAAACAACTTCATGATGTGTACATTCAACTGACAGGGTTGAACCTTCCTTTGGGTAGAG
CAGTTTTGAAACAGTGTTTTGACAGAATCCGCATGTGGATATTTGGAGCTCTTTGTAGCGTT
CATTGGAAATGGGAATATCTTCACTTGAAAACTGGACAGAAGCATTC
Monomer type t13

TCAGAAACTGCTTTGTGTTGTGTGCACTTGACACACAGAGTTGAACCTTTCTTTTGACAGAG
CACTCTTGAAACACTCTTTTTGTAGATTTTGTAAGTGTTCATTAGGAGCGCTTTGGAGCCTA
CTGTGGAAAAGGAAACAAATTCACATAGAAACTAGACAGAAGCATTC

Monomer type t14

TCTGAAAGTTATTTATGATGTGTGCTTTCAAATCACAGAGTTCACCCTTCCTTTTGAAAGAG
CAGTTTTGAAACACTCTTTTTAAAATATCTGCAAGTGGATATTTGGAACTCTTTGAGGCCTT
CGTTGGAAACGTGAATATCTTCCCATAAAAACTAGACAGAAGCATTC

Monomer type t15

TGTGAAACTGCTTTCTGTAGCGTGAATACAACCCACAGAGTTGAACACTTCTTTTGAAAGAG
CAGTTTTGAAACACTCTTTTTGTAGTATCAGCAATTGTTCATTAGGAGTGCTTTGAGGCCTA
TTGTGGAAGAGGAAATATCTTCACATAAAATCCAGACAGAAACATTC

Monomer type t16

TCAGAAACATCTTTATGATGTGTGCATTCAACACACAGTGTTGAAACTTCCTTTTGATGGAG
CAGTTTTGAAACAGTGCTTTTGCCGAATCTGCAAGTGGATATTTGGAGAACTTACAGGCCAT
CGTTGTAAAAGGGAATACCTTCAAATAAAAAGTAGACAGAAGCGTTC

Monomer type t17

CCTGAAATTGCTTTGTGTTGTATGCATTCAACTCACAGAGTTGAATCTTTCTTTTGATAAAG
CAGTTTTGAAACACTCTTTTTGTAGAGTCTAAAAGTTTTCCTTTGGTGCGCTTTGAGACCTA
TGGTGGAAAAGGAAATATTTTCACATAAATACTAGACAGAAGCATTC

Monomer type t18

TCAGAAAAATCTTTATGATGTGTGCATTCAACTCAGACAGATAAGCCTTCCTTTTGATAGAG
CAGTTTTGAAACAGTGTTTTTGCAGAATCTGCAAGGGGATATTTGGAGCTCTTGAGGCCTTC
GTTGGAAACGGGAATATCTTCACCAAAAAAGTAGACAGAAGTGTTC

Monomer type t10

TAAGAAACACCTTTATGATAAGGGCATACAACTAACAGAGTTGAACTTTCCTTTGGATACAG
CAGTTTTGAAACACCCTTTTTGTAGAATCTGCAAGCGTTCATTTGGAAGGCTTTGAGGCCTA
TGGTGGAAAACGAAATAGCTTCACATAAAAACTGGACAGAAGCAGTC

Monomer type t20

TCAGAAACACCTGTATGATGTGTGAATTCAACGCACAGAGTTGAACTTTCCTTTTCATGCAG
AAGTTTTGAAAGTGTCTTTTTCCAGTATTTGCAAGTGTTCATTAGGAGTGCTTTGAAGCCTA
CGGTGTAAAAGGAAACAACTTTACATAAAAACTAGACAGAAGCATTC

Monomer type t21

TCATAAATTGCTTTCTGATGTGTGTGTTCAACTCACAGAGTTGCACCTTTCCTTTGATAGAG
CCGTTTTGCAGCACCCTTTTTGTAGACTCTGCAAGTGTTCATTTGGAGTGCATTGAGGCCTA
CGGTGGAGAGGGAAATATCTTCACATGAAACCTAGACAGAAGCATTC

Monomer type t22

TCTGAAACTTCTTTATGATGTGTGCATTCAAGTCACAGAGTTGAACCTTCTTTTTCATGGAG
CAGTTTTGAAAGAGTCTTTTTACAAAATCTGCAAGTGGATACTTGCAGCTCTTTGAGGCCTT
CGTTGGAAACGGGAGTATCTTCAAATAAAAACTGGACAGAAGCGTTC

Monomer type t23

TCTGAAACTGCTTTCTGTTGTATGCGTTCAACTCACAGATTTGAATCCGTCTTTAGATAGAA
CAGTTTTGTAGCACTCTTTTTGTAGAATCTGCAAGTGTTCATTAGGTGTGCTTTGAGGCCTA
CAGTGGAAAAGGAAATATCATCACATAAAATCTAGACAGAAGCATTC

Monomer type t24

TCTGAAACTTTTTTATGATGTGTGCATTCAATTAACAGAGTTGAACATTCCTTTGGATAGAG
CAGTTTTGAAACAATATTTTTGCAGAATCTGCAAGTGGATATTTGGAGCTCTTTGAAGAGTT
CGTTGGAAACGGGACTATCTTCACATAAAAACTAGACAGAAGTGTTC

Monomer type t25

TCAGAAACTGCTTTGTGTTGTGTGCACTCAACACACAGAGCTCAACCTTTGTTTTGATAGAG
CACTTTTGAAACACTCTTTTTGTAGAATTTGCAAGTGTTCATTGGGAGAACTTAGAAGCCTA
CGGAGGAAAAGGAAACAACTTCACATAAAAACTACACAGAAGCATTC

Monomer type t26

TCTGAAATTCTTTCTGATGTGACCTTTCAACTCACAGAGTTCAAACTTCCTTTTGATGGAGC
AGTTTTTAAAGAGTCTTTTTGTGGAATCTGCAATTGGACATACGGAGCTCTTTGAGGCATTC
GTTGGAAACGTGAATATCTTCACATAAAAACTAGACAGAAGTGTTC
Monomer type t27
CAGAAGCATTCTGTGAAACAGCTTTGCATAGTGTGAAAACAACTCACAGGGTTGAACACTTC
TTTTCAAAGAGCAGTTTTGAAACCCTCTTTTTGTAGAATCTGCAATTGTTCAGTAGGAGCGC
TATGAGGCCAATAGTGGAAAAGGAAATATCTTCACATAAAATCCAGG
Monomer type t28
TCAGAAACATCTTTATGACGTGTGCATTCAACTCATAGAGTTGAACCTTCCTTTTGATAGAG
CAGTTTTGAAACCGTGTTTTGCAGAATCTGCAGGTGGATATTTGGAGCTTTTTCAGGCCTTC
GTTGGAAACGGGACTATCTTCAATAAAAACTAGA
Monomer type t29
TCACAAACTGCTTTGTGATGTGTGTGTTCACCTCACAGAGTTGAATCTTTCGTTTGATAGAG
CAGTTTTGAAACACCCTTTTTGTAGACTCTGCAAGTGTTCATTTGGAGCGCTTTGAGTCCTA
TAGTGGAAAAGGAAATATCTTCACATAAAAACCACACAGAAGCATTC
Monomer type t30
TCTGAAACTGCTTTATGATGTGTGCATTCAACTCACAGGGTTGAACCATCCTTTTCATGGAG
CAGTTTTGAAAGAGTCTTTTTGTCGAATATGCAAGTGGATATTTGGAGTTCTTTGAGGCCTT
CATTGGAAACGGGAATATCTTCACATAAAAACTGGACAGAAGCATCC
Monomer type t31
TAAGAAACTACTTTGTGTTGTGTGCACTCGACACACAGAGTTGAACCTATCTTCTGAAAGAG
CACTTTTGAAACACTCTTTTGGTAGAATTTGCAAGTGTTCATTAGGTGCGCTTTGAGGCCTA
TGGTGGAAAAGGAAATATCTTCACATAAAATCTAGACAGAAACATTC
Monomer type t32
TCTGAAAGTTCTTTATGAGGTGAGCTTTCAACTCACAGAAATCACCCTTCCTTTTGAAATAG
CCGTTTTGAAACACTCTTTTTTTGGAATCTGCAAGTGGATATTTGGAGCTCTTTGAGGCCTT
CATTGGAAACGTGAATATCTTCACATAAAAACTACACAGAAGCATTC
Monomer type t33
TGGGAAACTGCTTTGTGTAGTGTGAATCCAACTCACAGAATTCAACACTTCTTTTGAAAGAC
CAGTTTTGAAACACTCTTTTTGCAGAGTCTGCAATTGTTCATTAGGAGCGCTTTGAGGCCTA
TGGTGGAAAACGAAATATCTTCACATAAAATCCAGACGGAAACATTC
Monomer type t34
TCAGAATCATCTTTATGATGTGTGCATTCAACTCACAGAATTGAACACTCCTTTAGATAGAG
CACTTTTGAAACAGTGTTTTTGCAGAATCTGCAACTGATATTTTCAGCTCTTTCAGGCCTTC
TTTGGAAACGGGAATATCTTCACATAAAAGCTAGATAGAAGCGTTC
Monomer type t35
TCTGAAACTGCTTTCTGTTGTATGTATTCAACTCACAGATTTGAATATTTCCTTTCATAGAG
CAGTTTTGAAACACTCTTTTTGTAGAATCTGCTAGCGTTCATTTGGTCGGCTTTGAGTTCTA
TGGTGGAAAAGGAAATATCTTCACATAAAAACTAGACAGAAGCATCC
Monomer type t36
TCAGAAACATCTTTATGATGTGTGTATTCAACTCAGAGGGATAAGCCTTCCTTTTGATAGAG
TTGTTTTGAAACAGTGCTTTTGCAGAATCTGCAATGGATATTTGGAGCTCTTGAGGCCTTCG
TTGGAAACCGGAATATCTTCCCAAAAAAATTAGACAGAAGTGTTC
Monomer type t37
TCTGAAACTGCTTTGTGTTGTATGCATTCAACTCACAGAGTTGAATCTTTCTTTTGATAGAG
CAGTTCTGAAACACTTTTTTGTACAACCTGCAAGTGTTCATTAGGTGCACTTTGAGGCCTAT
GATGGAAAAGGAAATATCTTCACATAAAAACTAGACAGAAGCATTA
Monomer type t38
TAAGAAACAACTTTATGATGTGTACATTCAACTAACAGAGTTAAACCTTCCTTTGGATAGAG
CAGTTTTGAAACAGTGTTTTTGCAGGATCTGCAAGTGGATATTTTGAGCTCTTGAGGCCTTT
GTTGGAAACGGGAATATCTTCACATAAAAACTAGACAGAAATATTC
Monomer type t39

TCACATTCTGCTTTGTGTTGTGTGCACTCAGCACACGGAATTGAACCTTTCTTTTGAAACAG
TACTTTTGAAACACTCTTGTTGTAGAATTTGCAAGTGTTCATTAGGGGCGCTTTCAAGCCTA
TTGTGGAAAAGGAAAGAACGTCACATAAAAACTAGACAGAAGCATAC

Monomer type t40

TCTGAAAGTTATTTTGGATGTGTGCTTTCAACTCACAGAGTTCAACATTCCTTTTGAAAGAG
CAGTTTTGAAACATTCTTTTTATAGGGTCTGCAAGTGGATATTTGGAGCTCTTTGAGGCATT
CGTTGGAAACGTGAATATCTTCACATAAAACGTAGACAGAAGCATTC

Monomer type t41

TGTGAAACTGCTTTGTGTCGTGTGAATCCAACTCACAGAGTTGAACACTTCTTTTAAAAGAG
CAGTTTTGAAACCCTCTTTTTCTACAATCTGCAATTGTTCATTAGGAGCGCTTTGGGGCCTG
TGGTGTAAAACGAAGTATCTTCACATAAAATCCAGACAGAAGCATTC

Monomer type t42

TCGGAAACACATTTATGATGTGTGCATTCAACTCACAGAGCTGAACCTTCATTTTGATAGAG
TAGTTTTGAATCAGTGTTATTGCAGAATCTGCAAGTGATATTTGCAGCTCTTTCAGGCCTTC
GTTGGAAACGGGAATATCCTCACATAAAAACTAGACAGAAGTGTTC

Monomer type t43

TCGGAAACATCTTTATTATGTGTGCATTCAACTCACAGAATTGAACCTTCCTTTTGATAGAG
CAGTTTTGAAATAGTGTTTTTGAAAAATCTGCAAGTGGATATTGGCAGCTCTTTGAGGCCTT
CATTGGAAACCGGATTATCTTCACATAAAAACTAGACAGAAGCATTC

Monomer type t44

TCTGAAACTGCTTTGTGTTGTATGCATTCAACTCACAGGGCTGAATATTTGCTTAGATAGAG
TATTTTTGAAACACTCATTTTGTAGAATTTGTAAGTGTTCATTAGGTGCGCTTTGAGGCCTA
TGGTGGAAAAAGAAATATCTTCACATAAAAACTAGACAGAAGCATTC

Monomer type t45

ACAGAAGACTTCTCAAACAATCTTTATGATGTATGCATTCAATACAAACAGATGAGACTTCC
TTTTGATAGAGAAGTTTTGAAAGAGTGTTTTTGCAGAATCTGCAAGTGGATATTTGGAGCTC
TTGAGGAACTTCATTGGAAACGGGCATATCTTCACAAAAAAATAGACC

Monomer type t46

TCAGAATGCTTTGTGTTGTGTGTGTTCAACTCACAGTGTTGAACCTTTCTTTTGATAGAGCA
GTTTGAAACACCCTTTTTGTAGAATCTTCAAATGTTCATTTGGAGCACTTTGAGGCTTATGG
TGGAAAAGGAAATATCTTCACCTAAAAATTACA

Monomer type t47

TCTGAAACTTCTTTATGATGTGTGCTTTCAACTCACAGAGTTGAACCTTCCTTTTCATGGAG
CAGTTTTGAAAGAGTCTTTTTGCAGTATCTGCAAGTGGATATTTGGAGCTCTTTGAGGCCTT
CATAGGAAATGCAAATATCTTCACATAAAAGCTGGACAGAATCATTCTC

Monomer type t48

TCTGAAACTGCTTTGTGTTGTATGCATTCAACTCACAGAGGTGAACTTTTCTTTGCTAGAAC
GGTTTTGAAACACTCTTTTTGTAGAATCTGCAAGTGTTCATTCTGTGCGTTTTGAGGCCTAT
GGTGGAAGAGGAAATATCTTCACATAAAATCTAGACAGAAGCATTC

Monomer type t49

TAAGAAACAGCTTTATGATGTGTGCGTTCAACTAACAGAGTTGAACTTTCCTTTGGAAAGAG
CAGTTTTTAAACGGAGGTTTTGAAGAATCTTCCAGAGGATATTTGGAGTTCTTTGAGGTGTT
CGTTGGAAACGGGACTATCTTCACATAAAACTAGACAGAAGTGCTT

Monomer type t50

CCAAAAACTGCTCTGTGTTGTGTGCACTCAACACACAGAGTTGAACCTTTCTTTTGTTAGAA
CACTTTTCAAACACTCCTTTTGTAGAATTAGGAAGTGTACATTACGAGTGATTTGAAGAATT
CGGTGGAAAAGGAAACCACTTCACATTAAAACTAGACAGAAGCATTC

Monomer type t51

TCTGAAAATTCCTTATGATGTGTGCTTTCAACACAGACAGCTCAACCTTCCTTTTGATGGAG
CAGATTTGAAAGAGTCTTTTGCAGAATCTGCAAGTGGATATTTGGAGCTCTTTGAGGCCTTC
ATTGGAAACGTGAATATCTTCACATAAAAACCAGACAGCAGCATTT

Monomer type t52

TGTGTAACTGCTTTGTGTAGTGTGAATCCAACGCACAGATTTGAACACTTCTTTTGAAAGAG
CAGTTTCGAAACACTCTTTTTGTAGAATCTGCAGTTGTACATTAGGTGCGCTTTGAGGCCTA
TGGTGGAAAAGGTAATGTCTTCACAGAAAATCCAGACAGAGGCATTC

Monomer type t53

TCAGAAACATCTTTATGATGTGTGCATTCAACTCAGAGAGTTGAACCTTCCTTTTGATAGCA
GTTTTGAAACAGTGTTTTTGCAGAATCTGCATGTGGATATTTGGAGCTCTTTCAGGCCTTCG
TTGTAAACGTTAATATTTTCACATAATAACTAGACAGAAGCGTTC

Monomer type t54

TCTGAAACTGCTTTGTGTTGTATGCATTCAACTCACAGAGTTAAATCTTTCTTTTGATTGAG
CAGTTTTGAAACACTCTTTTTGTAGAATCTGCAAGTGTTCATTATGTGTGCTTTGAGGCGTA
TGTTGGAAAAGGAAATATCTTCACATAAAAACTAGACAGAAGAATTC

Monomer type t55

TCAGAAACATCTATATGATGTGTGCACTCAACCCAGTCAGATAAGCCTTTCTTTGGATAGAG
CAGTATTGAAAAAGTATTTTTGCACAATCTGCAAGTGGATATTTGGAACTCTTGATGCCTTC
ATTGGAAACGGGAATATCTTCACAAAAAAACGAGACAGAAGTGTTC

Monomer type t56

TCAGAAACTTCTTTGTGATGTGTGTGTTCAACTCACAGATTTGAACATTTCCTTTGACAGGG
CAGTTTTGAAAAACCCTTTTTGTAGAATCTGCAAGTGTTCACTCACAGCGCTTTGAGGTCTA
TGGTGGAAAAGTAAGTATTTTCACATAAAAAATTGACAGAAGCATTC

Monomer type t57

TCAGAAACATCTATGTGATGTGTGCACTCAACCCAGACAGATAAGCCTTTATTTGGATATAG
CAGTATTGAAAAGGTATTTTTGCACAATCTGCAAGTGGATATTTGGAGCTCTTGAGGCCTTC
GTTAGAAACGGGAATATCTTCACATAAAAACTGGACAGAAGCATCC

Table S2. Canonical 7mer consensus sequence.

Monomer type t1
TCAGAAACTTCTTTGTGATGAGTGCATTCTACTCACAGCGTTGAAGCTTACTTTTGATAGAG
CAGTTTTGAAACACTCTTTTGGTAGAATCTGCAAGTGGATATTTGGAGCGCTTTGAGGCCTA
TGCTGGAAAAGGAAATGTCTTCACATAAAAACTAGACAGAAGCATTC
Monomer type t2
CAGGAAACTTCTGTATGACAAGTGCATTCAACTCACAGACTTGAACGTTCCTTTTGATAGAA
CTCTTTTGAAACACTCCTTTTGTAGAATTTGCAAGTGTTTATTTGGAGCGCTTTGAGGCCTA
AGCTGGAAAAGGAAATATCTTCACGTAAGAACTAGACAGAAGCATTC
Monomer type t3
TCAGAAACTTCTTTCTGATGAGTGCATTCAACACACAGACTTGAACCTTGCTTTTGAAAGAG
CAGCTTTGAAACACTCTTTTCGTAGAATCTGCAAGTGGATATTTGGAGCGCTTTGAGGCCTA
TGCTGGGAAATGAAATCTCTTCACCTAAAAACTAGACAGAAGCATTC
Monomer type t4
TGAGAAACTTCTGTGTGATGAGTGCATTCAACTCACAGACTTGAACGTTCCCTTTGATAGAA
CAGTTTTGAAACACTCCTTTTGTAGAATCTGCAAGTGTTTATTCGGAGCACCTTGAGGCCTA
TGTTGGAAAAGGAAATATCTTCACATAAAAACTAGAGAGAAGGATTC
Monomer type t5
TCAGAAACTTCTTTGTGATGAGTTCATTCAACTCACAGAGTTGAACCTACCAATTGATAGAG
AAGTTTGGAAACACTCTTTTTGTAGAATCTGCAAGTGGATATTTGGAGCCGTTTGAGGCCTA
TGCTGGAAAAGGAAATATCTTCACATAAAAACTGAACAGAAGCATTC
Monomer type t6
TCAGAAACTTCTTTGTGTTGAGTGCATTCAACTCACAGACTTGAACTTACCTATAGAAAGAG
CAGTTTTGAAACACTCTTTTGGTAAAATCTGCAAGTGGTTATTTGGAGCACTTCGAGGCCTG
TGCTGGGATATGGAATATCTTCACCTAAAAACTACAGAGAAGCATTC
Monomer type t7
TCAGAAAGTTCTTCACGATGCATTCATTCAACTCACAGAGTTGAACCTTCCTTTTGATAGAA
CAGTTTTGAAACACTCTTTTTGTAGAATCTGCAAGAGGATATTTGGAGCGTTTTGAGGCCTA
TGCTGGAAAAGGAAATATCTTCACATAAAAACTAGACAGAAGCATTC

Table S3. Canonical 18mer consensus sequence.

Monomer type t1
TGAGAAACTTCCCTGCGCTATTTGCATTCAACTCACACTCTTGAACCTTCTTTTTGAAAGAG
CAGATTTGAAACACTCTTTTTGTAGAAACTGCAAGTGCATATTTGGAGTGCTTTGAGGCCTA
TGATGGAAAAGGAAATATCCTCACAGAAAAATTAGACAAAAGCATTC
Monomer type t2
AAAGAAATATCTTCACATAATACCTAGACGGAAGCATTCTCAGAAACTTCTCTGTGATGAGT
CATTTATGAAACACTTTCTTTGAAGAATCTATAAGAGATTATTTGGAGCTCATTGAGGCCTA
TGCTGGAAAAAGGAATATCTTCATATAAAAACTAGACAGAAGCATTC
Monomer type t3
TCCGAAACTTCTTTGGGATGAATGCATTCAGCTCACAGAGTTGAACCTTGCCTTTGATAGAA
CAGTTTTGAAACACTCTTTTTTTAGAATCTGCAAGTGGATATTTGGAGCGCATTGGGACCTA
TGCTGGAA
Monomer type t4
TCAGAAACTTCCCTTTGCTTTATGCAGTCAACTCACAGACTTGAAACTTCCTTTTCAAAGAG
CAGATTTTAAATACTCTTTTTGTAGAAACTGCAAGTGGATATTTGGAGCGCTTTGAGGCCTA
TGCTGGAAAAGGAAATATCCTCACATAAAAACAAGACAAAAGCATTC
Monomer type t5
TCAGAGACTTCCTTGTGCCTTATACATTCAATTCACAGGCTTGAACCTTCTTTTTGGTAGAG
CAGTTTTGAAACACCCTTTTTGTAGACTCTGCAAGTGGATATTTACAGCGATTTGAGGCCTA
CAGTGAAAAAGGGGATATCTTCACATAAAAACTAGATAGAAGCATTC
Monomer type t6
TCAGAAATTTCTTTGTGATGAGTGCGTTCAACTCACAGGGTTGAACTTTCCTTTTGATAGAA
CAGTTTTGAAACACTCTGTTTGTAGAATTTGCAAGTGGATATTTGGAGCGATTTGAGGCCTA
TGCTGCAAAAAGGGAATATCTTAACATAAGAACTAGACAGAAGCATTC
Monomer type t7
TCAGAAACATTTTTGTGATGAGTGCATTCAACTCTGAGACTTGAACATTCCTTTTGAAAGAG
CAGTTTTGAAACACTCTTTTAATAGAATCTACAAGTGGGTATTTGGAGCAATTTGAGGCCTA
TGCTGGAAAAGGGAATATCTTCACATAAAAATTAGACAGAAGCATTC
Monomer type t8
TCAGACACTTCTCTGTGATGGGCGCATTCAGCTCACTGAGTTCAACCTTCCTTTAGGTAGAA
TAGTTTTGAATCACTCTCTTTGTAGAATCTACTATCGATTATTTGGAGCGCATTGAGGCCTA
CGATGGAAAAGGGAATATCTTCCCATAAAAACTAAGCAGAATCATTC
Monomer type t9
TCAGAAACATTTATGTGATGAGTGCTTTCAACTCAGAGACTTGAACATTCCTTTCGATAGAG
CAGTTTTGAAACACTCTTTTTGTAGAATCTGCAATTGGATATTTGGAGTGCTTTGAGACCTA
TGCTGGAAAAGGAAATATCTTCACATAAAAATTAGACAGAAGTATTC
Monomer type t10
TCAGACACTTCTCTGTGATGAGTGCATTCGACTCACAGAGTTCAACCTTCCTTTTGGTAGAA
CAGTTTTGAATCACTCTCTTTCTAGGACCTACAGTAGATTATTTGGAGCTCCTTTAGGCCTA
TGATGGGAAAGGGAATATCTTCCCATAAAAACTAAGCAGAATCATTC
Monomer type t11
TCTGAAACTTCTTTGTGATGAATGCATTCAGCTTACAGAGTTGAACTTTCCTTTTGATAGAA
CAGTTTTGAAACACTCTTTTTGTAGAATCTGCAAGTGGATATTTGGAGAGCTTTGAGACCTA
TGCTGGAAAAGGAAATATCTTCACATAAAAATTAGACAGAAGCATTC
Monomer type t12
TCAGAAACTTCAGTGTGCGTTATGCATTCAACTCACAGACTTGAACATTCCTTTTGAAAGAG
CAGATTTGAAAAACTCTTTTTGTAGAAATTGCAAGTGGATATTTGGAGTGGTTTGAGGCCTA
TGCTGGAAAAGGATATATCCTCACATAAAAACTAGACAAAAGCATTC
Monomer type t13

TCTGAAACTTCTTTGTGATGCGTGCATTCATCTAACAGACTAGAAACTTCCTTTTGATAGAG
CAGTTTTGAAACACCCTTTTTGTAGAATCTACAAGTGGATATTTGGAGCGATTTGAGGCCTG
TGGTGTAAAAGGGAATATCTTCACATAAAAACTAGAAGGAAGCATTC
Monomer type t14
TCAGAAACATCTCTATGATGAGCGCATTGTACTCACAGACTTGAACTTTCTTTTTGATAGAG
CAGTTTTGAAACACTCCTTTTGTAGAATGTGCAAGTGGATATTTGGTGTGCTTTGAGGCCTA
ATCTGGAAAAGGAAATATCTTCACATAAAAACTAGACAGAAGCATTC
Monomer type t15
TCACAGAGTTCTTAGTGATGAGTGCATCCAACTCACAGACTTAAACTTTCCTTTTGATATAG
CAGTTTTGACACACTCTTTTTGTAGAATCTGCAAGTGGATATTTGGAGCGCTATGAGGCCTA
CGCTGTAAAAGGAAATATCATCACATAAAAACTATACAGAAGCATTC
Monomer type t16
TCTGAAACTTCTTTGCGATGAGTGCATTCAACCCACAGAGTTGAAACCTCCTTTTTTTTAGA
ACTGTTTTGTAACACTCTCTTTGTAGAATCTGAAAGTGGATACTTGGTGCGCTTTGAGGCCT
ATGCTGGAAATGGAAATATGTTCTCATAAAAACTAAGCAGAAGCATAC
Monomer type t17
CCAAAAACTTCCCCGTGCTTCATGCATTCAACTCACAGACTTGAACCTTCCTTTTGATAGAG
CAGTTTTGAAACACTCTTTTTGTAGAATCTGCAATTGGATATTTGGAGCGATTTGAGGCCTA
TGATGAAAAAGGGAATGTCTTCACCTAAAAACTAGACAGAAGCATTC
Monomer type t18
TCAGAAATTTCTTTGTGATGTGTGCATTTAATTCACCGAGTTGAACTTTCCTTTTGATAGAA
CAGTTTTGAAACAGTCTTTTTGTAGAATCTGCAAGTGGATATTTGGAGCATTTTGAGGTCTA
TGCTGGAAAAGGAAATATCTTCACATAAAAACTAGACAGAAGCATTC
Monomer type t19
TCAGAAATTTCTGTGTGATGAGTGCATTCAACTCACAGGGTTGAACTTTCCTTTTGCTAGAA
CAGTTTTGAAACATTCTTTTTGTAGAATCTGCAAGTGTGTATTTGGAGCGATTTGAGGCCTA
TGCTGCAAAAAGAGAAATCTTCACATAAAAACTAGACAGAAGCATTC
Monomer type t20
TCAGAAATATTTTTGTGATGAGTGCATTCAACTCACAGCCTTGAACCTTCCTTTTGATAGAG
TAGTTTTGAAACACTCGTTTTGTAGAATCTGCAAGTGGATATTTGGAGTGCTTTGAGGTCTA
TGCTGGAAAAGGAAATATCTTCACATCAAAACTAGACAGAAACATTC
Monomer type t21
ACAGAAACTTCTCTGTGATGAGTGCATTCACCTCACAGAGTTCAACCTTCCTTTTGGTAGAA
CAGTTATGAAACACTCTCTTTGTAGAGTCTACAAGAGATTATTTGGAGCTCATTGAGGCCTA
TGCTGGAAAAGAAAGTATCTTCCCGGAAAAACTAAGCAGAATCATTC
Monomer type t22
TCCGAAACTTCTTTGTGATGAATGCATTCACCTCACAGAGTTGAACCTTCCCTTTGATGGAA
CAGTTTTGAAACACTCTTTTTGTAGAATCTGCAAGTGGCTATTTGGAGCGCTTTGAGGCGTA
TGCTGGAAAAGGAAATATCTTCACATAATAAATAGACGGAAGCATTC
Monomer type t23
TCAGAAACTTCCCTGTGCCGTATGCATTCAACTCACGGACTTAAAACTTCCTTTTGAAAGAG
CAGATTTGAAACACTTTTTTTGTAGAAACTGCAAGTGGATATTTGGAGCGCTTTGAGGCCTA
TGCAGGAAAAGGAAATATCCCCACATAAAAAGTAGACAAAAGCATTC
Monomer type t24
TCCGTATTTTCTTTGTGATGTGTGCATTAAACTAACAGAGTAGAACCTTCCTTTTGATAGAG
CAGTTTTGAAACCCTCTTTTTGTAGAATCTGCAAGTGGATATTTGGAGCGATTTGAGGCCTA
TGGTGAAAAAGGGGATATCTTCGCATAAAAACTAGATAGAAGCATTC
Monomer type t25
TCAGAAACTTCTTTGTGATGAGTGCATTGTACTCACAGACTTTAAATTTCCTTTTGATAGAG
CAGTTTTGAAACAGTCTTTTTGTAAAATATGAAAGTGTATATTTGGTGCGATTTGAGGGCTA
AGATGGAAAAGGAAATATCTTCACATAAAAACTGGACAGAAGTATTC
Monomer type t26

TCAGAAACTTCTCTGTGTTTTATGATTTCAACTCACAGAGTTGAACCTTCCTTTTGATAGCG
CAGTTTTGAAACACTCTTTTGTAGAATCTGCAAGTGGATATTTGGAATGCTTTGAGGCCTAT
GCTCGAAAAGGAAATTTCTTCATATAAATACTAGACAGAAGCATGC

Monomer type t27

TCAGAAACTTCTTTGTGATGTGTGCACTCAACTCACAGATTTGAACCCTGCTTTTCAAAGAG
CAGTTATGAAACACCCTTTTTGTAGGATCTGCAGGTGGACATTTTTGGCGCTTTGAGGCCAA
TGCTGGAAAAGGAAATATGTATACATAAAAACTAAACAGAAGCATTC

Monomer type t28

TCAGAAACTTCCCTGTGCTTTATGAATTCAACTCACAGACTTGAACCTTCCTTTTGATAGAA
CAGTTTGGAAAAACACTTTTTGTAGAATCTGGAAGTGGATATTTAGAGCGCTTTGAGGCCTA
TGCAGGAAAAGGAAATACCATCACATAAAAACTAGACAGAAGTATTC

Monomer type t29

TCAAAAACATCTTTGTGATGTGTGCCTTCAACTCACAGAATTGAAACTTCCTTTTGATAAAG
CAGTTTTGAAACACCATATTGTAGAATCTGCAAGTGGATAGTTTTAGCACTTTGAGGCCTAG
CCTGGAAAAGGGTATATCTTCACATAAAAACTGGACAGAAGCATTC

Monomer type t30

TCAGAAACTTCTTTGTGATGAGTGCATTCAACTCACAGTGTTGAAAGTTCCTTTTGATAGAA
CAATTGTGACACAATCTTCTTATAGAATCTGCAAGTGGATATTTGGATTGCTTGAGGCCTAT
GCTGGAGTAAGAAATAATTTCACATAAAAACTATATTGAAGCATTC

Monomer type t31

TCAGAAACTTCTGTGGGATCAGAACGTTCAACCAACAGAGTTGAACCTTCCTTTTGATAGAC
TAGTTTTGAAACACACTTTTTGTAGAATCTGCAAGTGGATGTTTGGAGGGCTTTGAGGCCTA
TGCTGGAAAGAAAATATCTTCACCTAAAAACTAGACAGAAGCATTC

Monomer type t32

TCAGAGACTCCCCTGTGCTTTATGCATTCAAATCACAGGCTTGAACCTTCCTTTTGATAGAG
CAGTTTTGAACCACTCTTTTTGTAGAATCTGCAAGTGGATATTTGGAGTGATTTGAGGCCTA
TGATGAAAAAGGAAATATCTTCACCTAAAAACTAGACAGAAACATTC

Monomer type t33

TCAGAAATTTCTTGGTGATGAGTGCATTCAACTCACAGGTTTGAACTTGCCTTTTGATAGAA
CAGCTTTGAAACACTCTGTTTGTAGAATTTGCAAGTGGATATTTGGAGAGATTTGTGGCCTG
TGCTGCATAAAGGGAATATCTTAACTTTAGAACAAGACAGAAGCATTC

Monomer type t34

TCAGAAATTACTTCGGGATGAGTGCATTCAACCCAAAGAGTTGAACCTTCCTTTTGATAGAA
CAGTTTTGAAACACTTTTTTTGGAGAATCTGCAAGTGGATATTTGGAGCAATTTGAGGCCTA
TGCTAGAAAAGGAAATATCTTCACATAAAAACTAGACGGAAGCATTC

Monomer type t35

TGCGATACCTTTTTGTGATGAGTGCATTCAACTCACAGACTTGAACATTCCTTTCGATAGAG
CAGTTTTGAAACACTCTTTTTGTAGATTCTGCAAGTGGGTATTTTGAGCGCTTTGAGGTCAA
AGATGGAAAAGGAAATATCTTCATATAAAAACTAGACAGAAGCATTC

Monomer type t36

TCAGACACTTCTCTGTGATGAGTGCATTCAACTCACAGAGTTCAACCTTCCTTTTTGTAGAA
CAGTTATGAAACACTCTCTTTGTAGAATCTACAAGGGATCATTTGGAGCACATTAAGGCCTA
TGCTGGAAAAGGAAGTATCTTCCCATAAAAAAAAAAGCAGAATCATTC

Monomer type t37

TGAGATACTTTTTTGTGATCAGTGCATTCGACTCACAGACTTGCACCTTCCTTTTGATAGAG
CAGTTTTGAAACACTCTTTTTGAAGAATCTTCAAGTGGATATTTGGAGCGCTTTGAGGCCTA
TGCTGGAAAAGGAAATATCTTCACATAAAAACTAGACAGAAGCATTG

Table S4. Canonical 14mer consensus sequence.

Monomer type t1
TGTGAAACTGGTTTGTGTAGCGTGAATCCAACTCACAGAGTTGAAAAACTTTTTCTGAAAGA
GCAGTTTTGAAATATTCTTTGTAGAATGTGCAATTGTTCATTAGGAGCACTTTGAGGCCTAT
GTTGGAAAAGGAAGTATCTTCACAAAAAATCCAGACAGAAGCATTC
Monomer type t2
TCAGAAACAATTTTTTGATGTGTGCATTCAACTCACAGAGTTGAACCTTCTTTTTCTTAGAG
CAGTTTTGAAACAGTGTTTTTCCAGAATCTGCAAGTGGATATTTGGAGCACTTACAGGCCAT
CGTTGGAAATGGTAACATCTTCAAATAAAAACTAGATGGATGCATTC
Monomer type t3
TCAGAAACTGCTTCGTGTTGTATGCCTTCAACTCACAATGTTGAATCTTTCTTTTGATAGAG
CAGTTTTGAAACACTCTTTTTTTGTAGAATCTGCAAGTGTTCATGGGGTGTGCTTTGAGGTC
TATCGTGGAAAAGGAAATATCTTCATATAAAAACTAGACAGAAGCATTC
Monomer type t4
TCAGAAACACCTTTATGGTGTGTGCATTGAACTCAGAGAGATAATCCTTCCTTTTGATAGAG
CAGTTTTGAAACAGTGTTTCTGCAGAATATGCAAGTGGACATTTGGATCTCTTGAGGCCTTC
GTTGGAAACCAGAATATCTCACACAAAAAAACTAGACAGAAGTGTTC
Monomer type t5
TCAGAAACTGCTTTGTGATGTGTTTCTTCAACCCACAGAATTGAACATTTCCCTTGATAGAA
CAGTTTTGAAACACCCTTTTTGTAGAATCTGCAAGTTTTCTTTTGGAGGGCTTTGTGGCCTA
TGGTGGAAAAGGAAATATCTTCATATAAAAACCAGACAGAAGGTTTC
Monomer type t6
TCTGAAACTTCTTTACGATGTGTGCATTCAACTCACAGACTTGAAACTTCCTTTTCGTGGAG
CAGTTTTCAAAGCGTCATTTTGCAGAATCTGCAAGTGGATATTTGTAGCTCTTTCAGGCTTT
CGTTGGAAACGGGAATATCTTCACATAAATACTGGACAGCAGCATTC
Monomer type t7
TCTGAAACTACTTTCTGTTGTATGCGTTCGTCACCCAGAGTTGAATCTGTATTTTGATAGAA
CAGTTTTGGAAAAGTCTTTTTGTAGAATCTGCAAGTGTTCATTTGGTATGCTTTGAGGCCAA
TGGTGGAAACGGAAATACTTTCACATAAAATCTAGACAGAAGCATTC
Monomer type t8
TCTGAAATTTCCTTATGATGTGTGCATTCAACAAACAGAGTTCAACCTTCCTTTGGGTAGAG
CAGTTTTGAAATAGTATTTTTGCAGAATCTGCATAAGGATATATATGGAGCTCTTTGAAGCG
TTCGTTGGAAACGGGACTATCTTCACATAAAAACCAGACAGAATTGTTC
Monomer type t9
TCAGAAACTGCTTTGTGTTGCGTGCATGCAACACACAGAGTTGAACCTTTCTTTTGATAGAG
CACTTTTGAAACACTCTTTTTGTAGAATTTGCAAATGTTCATTAGGAGCGCTTTGAAGCCTA
TGGTGGAAAAGGAAACAACTTCACAGAAAAACTAGACAGAAGCATTT
Monomer type t10
TCTGAAACTTCTTTAAGATGTGTGTATTCAACTCAGAGACTTGAACCTTCCTTTCGATGGAG
CAGTCTTGAAAGAGACTTTTCGCAGAATCTGCCAGTGGATATTTGGAGCTCTTTGAGACCTT
CGTTGGAAACGTGAATATCTTCACATAAAAACTAGACAGAAGTGTTC
Monomer type t11
TCTGAACCTGCTTTGTGTTGTACACATTCAACTAACAGAGTTGAATCTTTCTTTTAATACAG
CAGTTTTGAAACACTTTTTTTTATAGAATCTGCAAGTTTTCATTCGTTGTGCTTTGAGGCCA
ATGGTGGAAAAGGAAATATCTTCACATAAAATCTAGACAGAAGCATTT
Monomer type t12
TAAGAAACTCCTTTATGACGTGTGCATTTAACTAACAGAGTTGAATCTTCCATTGTTTGAGC
AGTTCTGAAACAGTGTTTTTGCAGAATATGCATGTGGATATTTGGAGCTTTTTGAGGCGTTC
GTTGGAAATGGTACAATCTTCACATAAAACTAGACAGAAGTGTTC
Monomer type t13

TCAGAAACTTCTTTGTGTTGTGTGCACTCAACACACAGAGTTGAACCTTTCTTTTGATAGAG
CACTTTTCAAACACTCTTTTGGTAGAATTTGCAAGTGTTCATTTGGAGGGAATTGAAGCCCA
CGGTGGAAAAGGAAACAATGTCACATAAAAACATGACGGAAGCATTC

Monomer type t14

TGTGAAAGTTATTTATGATGTGTGCTTTCAACTCACAGAGTTCAACCTTCCTTTTGAAAGAG
CAGTTTTGAAACATTCTTTTTATGTTATCTGCAAGTGGATATTTGGAGCTCTTTGAGGCCTT
TGTTGGAAACGTGAATATCTTCACATAAATACTAGACAGAGGTATTC
