# Supplementary material for: Novel Cascade Alpha Satellite HORs in Orangutan Chromosome 13 Assembly: Discovery of the 59mer HOR—The largest Unit in Primates—And the Missing Triplet 45/27/18 HOR in Human T2T-CHM13v2.0 Assembly
Source: Int J Mol Sci. 2024 Jul 11;25(14):7596. doi: 10.3390/ijms25147596 (PMC11276891; doi:10.3390/ijms25147596)

Fig. S3. (separate file) Cascading interspersed 18/27/45mer alpha satellite HOR alignment. Start position 7,917,393 bp and end position 8,334,639 bp in GCF_028885655.2. The numbers on the left side indicate the initial position of the first monomer in each HOR copy.

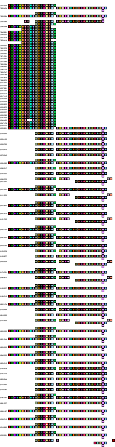

Supplement: Supplementary file 1 [file ijms-25-07596-s001.zip › Supplementary Figure S3.pdf]
