# Supplementary figures and images for: Novel Cascade Alpha Satellite HORs in Orangutan Chromosome 13 Assembly: Discovery of the 59mer HOR—The largest Unit in Primates—And the Missing Triplet 45/27/18 HOR in Human T2T-CHM13v2.0 Assembly
Source: Int J Mol Sci. 2024 Jul 11;25(14):7596. doi: 10.3390/ijms25147596 (PMC11276891; doi:10.3390/ijms25147596)

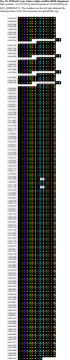

Supplement: Supplementary file 1 [file ijms-25-07596-s001.zip › Supplementary Figure S4.pdf]
